# Surface Characterization and Tribological Performance of Anodizing Micro-Textured Aluminum-Silicon Alloys

**DOI:** 10.3390/ma12111862

**Published:** 2019-06-09

**Authors:** Luanxia Chen, Zhanqiang Liu, Bing Wang, Qinghua Song, Yi Wan, Long Chen

**Affiliations:** 1 School of Mechanical Engineering, Shandong University, Jinan 250061, China; sduchenluanxia@gmail.com (L.C.); sduwangbing@sdu.edu.cn (B.W.); ssinghua@sdu.edu.cn (Q.S.); wanyi@sdu.edu.cn (Y.W.); chenlong@sdu.edu.cn (L.C.); 2 Key Laboratory of High Efficiency and Clean Mechanical Manufacture of MOE/Key National Demonstration Center for Experimental Mechanical Engineering Education, Jinan 250061, China

**Keywords:** micro-textures, anodizing, superficial hardness, bearing area ratio curves, friction

## Abstract

Eutectic aluminum-silicon alloys present high frictional coefficient and a high wear rate due to the low hardness under sliding friction conditions. In this paper, the eutectic aluminum-silicon alloy was textured firstly by micro-milling operations. Then, the micro-textured specimen was subjected to anodizing to fabricate alumina films. The surface topography, surface roughness, and bearing area ratio of micro-textured and anodizing micro-textured specimens were measured and characterized. For the anodizing micro-textured specimens, the surface roughness and superficial hardness increase compared with those for micro-textured ones. Tribological tests indicate that anodizing micro-textured samples present lower friction coefficient of 0.37 than that of flat samples of 0.43 under dry sliding conditions. However, they exhibit higher friction coefficient at 0.16 than that of flat samples of 0.13 under oil-lubricated conditions. The difference between the friction coefficient of anodizing micro-textured and flat samples under dry and oil-lubricated conditions is ascribed to the influence mechanism of surface roughness, bearing area ratio curves, and its relative parameters on the tribological performance of testing samples. The dry sliding friction coefficient has a positive correlation with bearing area ratio curves, while they present negative correlation with bearing area ratio curves under oil-lubricated conditions. The synergy method treated with micro-milling and anodizing provides an effective approach to enhance the dry sliding friction property of eutectic aluminum-silicon alloys.

## 1. Introduction

As the versatile material in automobile and military engineering, aluminum-silicon alloys are attractive for their high strength to weight ratio, excellent castability, high thermal conductivity, good wear, and corrosion resistance. Depending on the silicon content, aluminum-silicon alloys are classified into three types, i.e., hypoeutectic, eutectic, and hypereutectic. However, eutectic aluminum-silicon alloys present high frictional coefficient and a high wear rate due to the low hardness under sliding friction conditions. Various approaches to enhance the friction property of eutectic aluminum-silicon alloys are listed as follows: Microstructural modification by rapid solidification, such as selective laser melting [1,2,3]; morphology modification; reinforcement modification [4,5]; alloying [6,7]; and surface modifications [8,9,10].

Surface micro-texturing performed as a surface modification approach is attractive for enhancing the frictional property of mechanical components. Micro-textures act as the entrapment of wear particles to reduce the abrasion wear under dry sliding conditions [11,12,13,14]. Under boundary and mixed lubrication, micro-textures perform as lubricant reservoirs to generate secondary lubrication [15,16]. Furthermore, micro-texturescanemerge the hydrodynamic pressure lubrication to improve load carrying capacity under full-film lubrication [17,18,19].

However, Houdková et al. [20] concluded that the positive effect of micro-textures on frictional behavior was observed only on the initial stage of friction under the high-loaded testing conditions. Micro-textures on Al-Sn-Si samples were worn out rapidly by the counterparts. They then had no influence on the frictional property of testing specimens. To date, nitrogen ion implantation, anodic oxidation, high-velocity oxy fuel spraying, physical vapor deposition (PVD), and laser cladding have been employed on metal alloys after micro-texturing to improve their antifriction property [21,22,23,24].

Anodic oxidation of aluminum alloys synergistic with surface micro-texturing technology has been investigated recently [25,26,27]. However, most of the recent researches have focused on the improved surface hardness, deposition of PTFE/MoS_2_ particles on porous anodic aluminum oxide film, and its tribological properties under dry sliding conditions [10,25,28,29]. The tribological property and surface topography changes ascribed to anodizing process were not well investigated under dry and oil-lubricated conditions. Surface topography of aluminum-silicon alloys was changed with anodizing. Most of the researchers neglected the impact of surface topography on the friction property.

The frictional resistance is proportional to the load, independent of the apparent contact area, and sliding velocity of sliding surfaces has been known for a long time [30]. However, at the constant load, friction coefficient is material-dependent and often found to take different values for different conditions (e.g., humidity, surface property) of the sliding surfaces [31]. A large number of experiments have shown that in the early stage of friction and wear, the friction coefficient is greatly affected by the surface property parameters. The surface property parameters will affect the real contact area of two contact surfaces. Two of the most important surface property parameters are surface roughness and topography. The effect of surface roughness on the tribological performance needed to be investigated for proper design of contact surface.

Sedlaček et al. [32,33] investigated the correlation between standard roughness parameters (skewness, kurtosis) and tribological behavior of contact surfaces. Pin-on-disc test results indicated that surfaces with higher *Sku* and negative *Ssk* values tended to reduce friction under dry and lubricated conditions. Standard surface roughness parameters *Sa* and *Sq* were not sufficient to determine the tribological performance of contact surfaces as they provided only a rough estimate of the roughness class. A higher surface roughness (*Sa*, *Sq*) generally resulted in lower friction but longer distances to reach steady-state conditions under dry sliding conditions. However, the friction coefficient was lower while the roughness (*Sa*, *Sq*) was low. Surface roughness parameters *Ssk*, *Sku*, and *Svk* were demonstrated to show a good correlation to the tribological performance of contact surfaces.

Zhu et al. [34] investigated the influence mechanism of morphological parameters on tribological behaviors based on bearing area ratio curve. They established functions correlating the dry or lubricated friction coefficient and the bearing area ratio curves. Forty-five steel disc samples were prepared using different grades of grinding, polishing, turning, and milling methods to obtain diverse surface topography parameters to verify the functions correlation attained.

**Figure 1:**
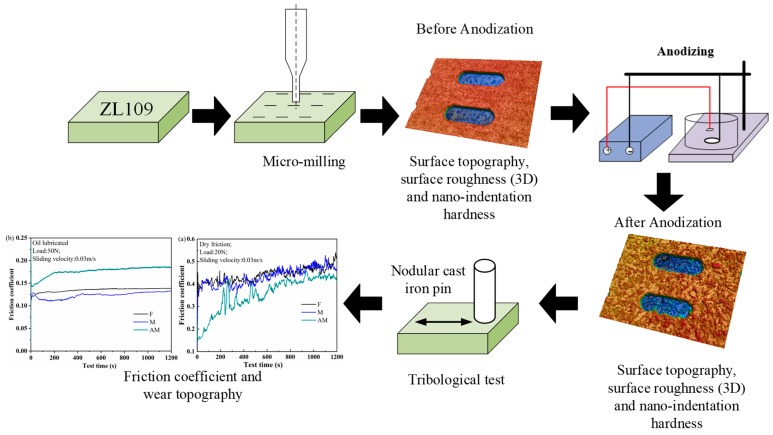
Framework structure of this study.

In the present paper, the impact of surface roughness on the friction property of eutectic aluminum-silicon alloys was investigated under dry and oil-lubricated conditions. The framework structure of this study is illustrated in Figure 1. The micro-milling process depended on forced material removal and was utilized to manufacture micro-textures on specimens. Then, the textured specimen was subjected to anodizing to fabricate alumina films. The phase composition and microstructure of the aluminum oxide films were investigated by X-ray diffractometer (XRD), scanning electron microscope (SEM), and energy dispersive spectroscopy (EDS) analyses. This sequential process is easy to implement with a high efficiency and low cost. Confocal laser scanning microscopy (CLSM) and SEM were employed to characterize the surface topography before and after anodizing. Nano-indentation tests were performed to measure the superficial hardness of alumina films. Frictional coefficients of testing specimens were reported to investigate the effect of surface roughness on frictional properties under dry friction and oil-lubricated conditions.

## 2. Materials and Experimental Setup

### 2.1. Preparation Process

#### 2.1.1. Materials

Commercially available, flat eutectic aluminum-silicon alloys samples (10 mm × 10 mm × 5 mm, ZL109, GB/T 1173-2013) were used for the substrate under dry friction conditions. Table 1 displays the chemical composition of ZL109. Firstly, the flat ZL109 samples and nodular cast iron pins were wet polished using #600 to #1200 waterproof emery papers. Then, they were polished with 5 μm diamond suspension to obtain the mean surface roughness *Sa* of 1.17 and 0.56 μm, respectively.

The five-axis CNC milling machine (Kern Micro 2522, Kern Microtechnik GmbH, Eschenlohe, Germany) with a maximum rotating speed of 50,000 rpm was employed to micro-mill the micro-textures on the ZL109. A 4-flute cemented carbide end mill of 6 mm diameter (MX430, NS TOOL, Tokyo, Japan) was used for cutting the plane to fabricate surfaces under the same cutting parameters. Then, the tungsten carbide flat micro end mill with 200 µm diameter (MSES230P, NS TOOL, Tokyo, Japan) was employed to fabricate the rectangle dimples with arcs on specimens. Cutting parameters of micro-milling experiments to fabricate micro-textures are listed in Table 2. Supporting material was employed to control burr formation in the micro-milling experiment [35]. After micro-milling experiments, the supporting material was removed ultrasonically in acetone. Moreover, surface topography of micro-textured specimens and polished flat specimens were characterized with CLSM. All the tested specimens were ultrasonically cleaned in acetone and ethanol baths for 15 min each.

#### 2.1.2. Anodization

The flat and micro-textured plate was degreased ultrasonically in ethanol and acetone for 10 min each. Then, the flat and micro-textured plates with a planar area of 100 mm^2^ were used as the substrate. The other surfaces were coated with resin to prevent from anodic oxidation. The anodization of ZL109 involved the surface pretreatment and anodic oxidation.

Before anodic oxidation, the flat and micro-textured plates were immersed into the alkaline electrolyte consisting of 20 g/L Na_3_PO_4_ and 5 g/L NaOH for 3 min at room temperature. This process was intended to remove the oxides from the ZL109 surface. Then, they were chemically polished in the solution containing aqueous HNO_3_ (50%, V/V), HF (5%, V/V) for 3 min to remove the black stains at 25 °C. The distilled deionized water was used to rinse experimental samples after each process.

The anodizing was performed in an electrolyte cell with flat and micro-textured ZL109 sheet as anode, lead sheet as cathode, and phosphoric acid solution (4 wt%) as electrolyte. The two electrodes were placed at a fixed distance of 10 mm. Anodization was carried out at the current density of 0.025 A/cm^2^ and a duration of 35 min. The electrolyte temperature during anodization was retained at 20 °C.

After anodizing, the surface topography of micro-textured specimens combined with anodizing was characterized by SEM (JSM-6610LV, Jeol, Tokyo, Japan) and CLSM (VK-X200 series, Keyence, Osaka, Japan). The phase constituents of ZL109 before and after anodizing were characterized by using an X-ray diffractometer (D8 Advance, Bruker AXS, Karlsruhe, Germany) with Cu Kα radiations (λ = 1.5406 Å), and the data were collected in the 2*θ* range from 20° to 80°.

### 2.2. Nanoindentation

The nanoindentation test was performed using a MML NanoTest TM nano-indenter (NanoTest TM, Micro-Materials, Wrexham, UK) to measure the hardness of ZL109, nodular cast iron, and aluminum oxide. A fixed maximum displacement indentation method with a Berkovich shaped diamond tip diameter of 50 nm was used in the nanoindentation test. In addition, the test parameters for nanoindentation are listed in Table 3. To avoid the substrate effect, the maximum indentation depth for the aluminum oxides was 240 nm, which should be less than 10% of the total film thickness (about 3000 nm) to meet the International Organization for Standardization ISO: 14577-1:2002E. 

### 2.3. Friction Tests

Dry sliding friction tests were performed using a pin-on-plate configuration (UMT-3, CETR, USA) in the linear reciprocating sliding mode with the normal load of 20 N and the sliding speed of 0.03 m/s. Under oil-lubricated conditions, the applied load was 50 N, and the sliding velocity was 0.03 m/s. The upper counter-body was a nodular cast iron pin with the diameter of 5 mm. The contact surface between nodular cast iron pins and plate was flat. Each friction test continued for 1200 s. Three identical samples were made to conduct the tribological tests to evaluate the frictional performance.

After the friction tests, EDS analysis was employed to examine elemental characteristics of the wear tracks of testing specimens. In this paper, the flat sample is denoted as F, the micro-textured sample proceeded is designated as M, the anodizing micro-textured specimen is named as AM.

## 3. Results and Discussion

### 3.1. Surface Topography and XRD Analysis

**Figure 2:**
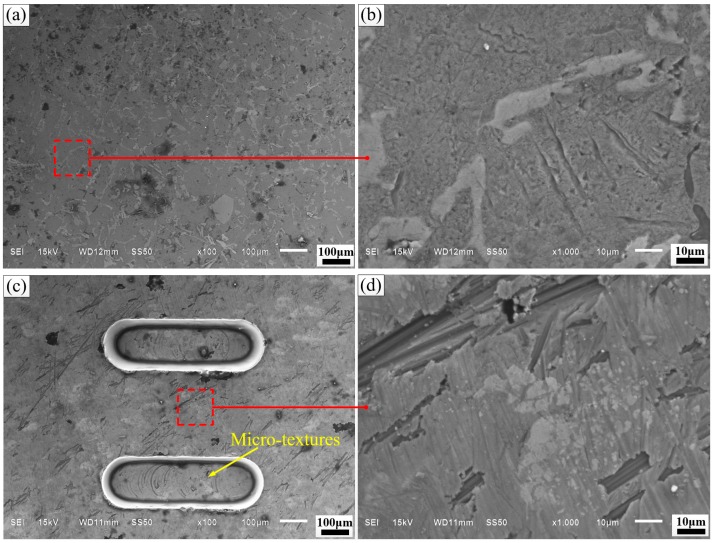

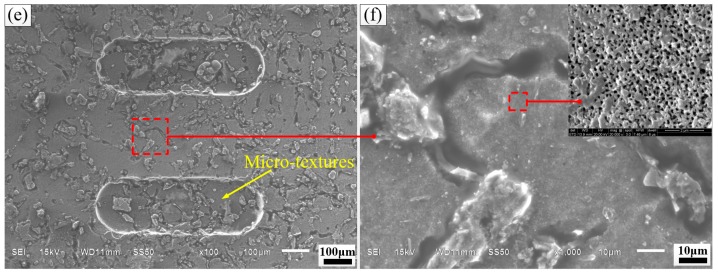
SEM and enlarged morphology of (**a**,**b**) flat; (**c**,**d**) micro-textured; (**e**,**f**) anodizing micro-textured specimens.

The surface morphologies of F, M, and AM specimens are characterized by SEM in Figure 2. Figure 2a,b depicts the surface morphology of flat specimens. Figure 2c,d illustrates the SEM topography of micro-textured specimens. Before anodizing, the length, width, and distance of rectangle dimples are 600, 200, and 500 µm. The depth is 45 µm, and the dimple area density is 22.2%. The surface micrograph of micro-textured specimens treated with anodizing is illustrated in Figure 2e,f. The anodized surface becomes rougher than the micro-textured one. Porous aluminum oxide films have been fabricated on the surface of ZL109 by anodizing. The phosphoric acid anodic film surface appears to be relatively uniform. The pores are of irregular shapes, and the array is regular [36].

**Figure 3:**
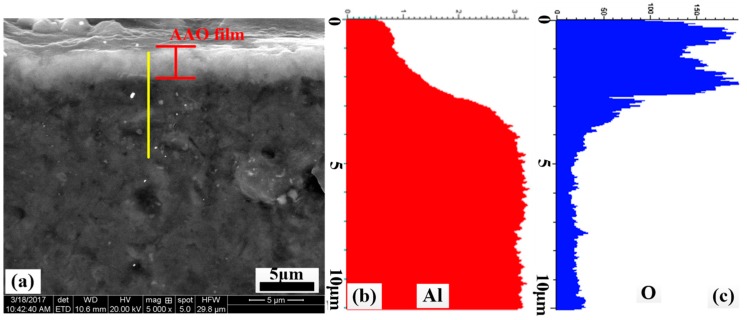
Cross-sectional SEM morphology of anodized (**a**) ZL109; and corresponding EDS line scan of (**b**) Al; (**c**) O element.

The cross-sectional SEM micrograph of anodized ZL109 is illustrated in Figure 3a. Alumina films are distributed evenly on ZL109 alloys with the thickness of 3 μm. The distribution of the aluminum oxide films’ elements is checked by EDS line scanning, and the results are presented in Figure 3b,c. As can be seen, the Al and O elements obviously diffuse on the ZL109. The aluminum and oxygen directly result from the growth of the anodic film.

**Figure 4:**
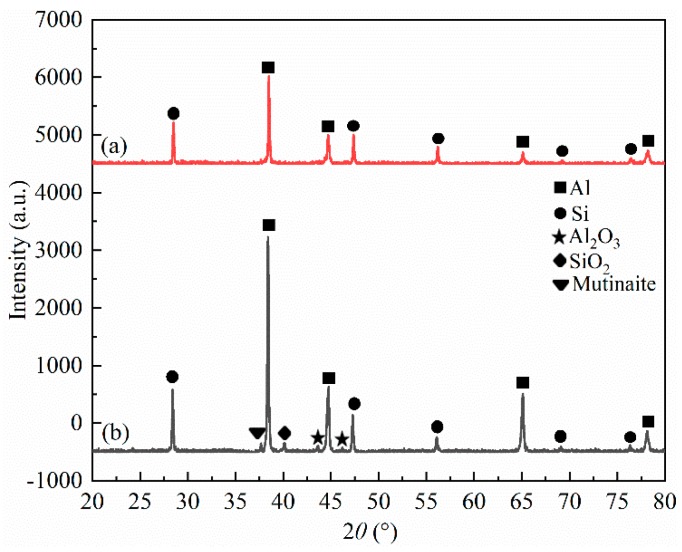
XRD for ZL109 (**a**) before; (**b**) after anodizing.

The XRD patterns of ZL109 before and after anodizing performed on the surface are shown in Figure 4. The XRD pattern of ZL109 before anodizing shows that peaks of Al and Si arise from ZL109 alloy matrix of the composites (Figure 4a). Figure 4b shows that the main phase constituent of the aluminum oxide films is Al and Si. Al and Si come from ZL109 alloy substrate. The aluminum oxide films consist of mutinaite (Al_2_O_3_·54SiO_2_), Al_2_O_3_, and SiO_2_ phase, which formed mainly in the reaction process of anodization.

**Figure 5:**
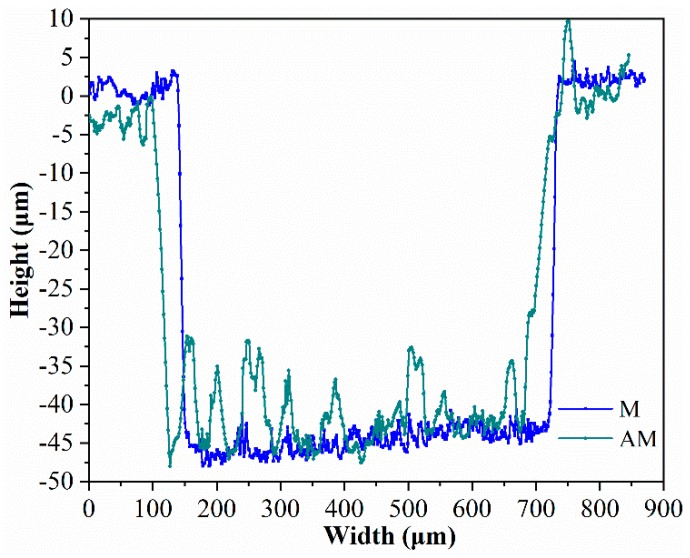
Cross-sectional view of a single micro-texture before and after anodizing.

Two-dimensional cross-sectional views of a single micro-texture along the long side are demonstrated in Figure 5. The length, width, and depth of micro-textures before and after anodizing are almost the same. Surface topography of micro-textures is changed through anodizing. The bottom topography of micro-textures after anodizing becomes rough compared with that of micro-textures before anodizing.

### 3.2. Surface Roughness and Bearing Area Ratio Curve

ISO 13565-2: 1997 defines a set of parameters, based on the linear material ratio curve, to be used for evaluating the valley suppressed roughness profile, which is based on a three-layer surface model, evaluating the peaks, the core, and the valleys separately. They are intended to aid in assessing the operational behavior of highly mechanically stressed surfaces.

The arithmetical mean height (*Sa*), root mean square height (*Sq*), skewness (*Ssk*), kurtosis (*Sku*), and other parameters for the samples studied in the research are summarized in Table 4. Samples were prepared using micro-milling and anodizing, which resulted in *Sa* values increased from 0.89 μm to 1.81 μm and different values of other parameters. Variable specimens with similar *Ssk* and *Sku* but different *Sa* and *Sq* values were prepared to investigate the effect of the *Sa* and *Sq* parameters on the tribological behavior of contact surfaces under dry and oil-lubricated conditions.

**Figure 6:**
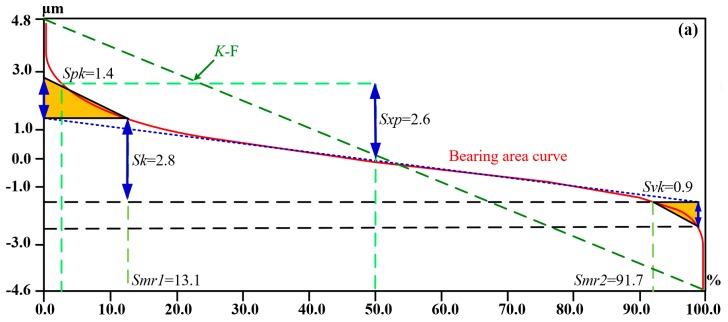

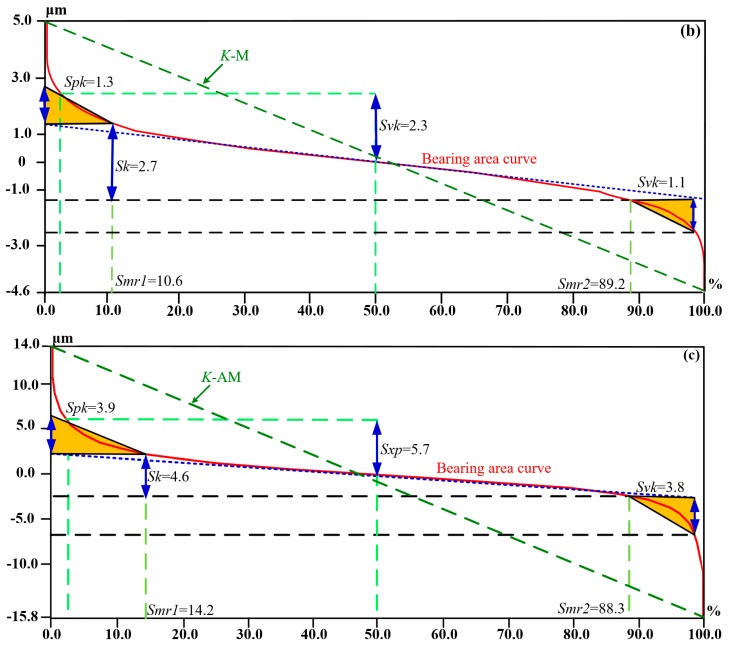
Bearing area curves of (**a**) flat; (**b**) micro-textured; (**c**) anodizing micro-textured samples.

The bearing area ratio curves of F, M, and AM samples are illustrated in Figure 6. The bearing area ratio curve is the cumulative curve of the distribution. The bearing area ratio curve is counted from the highest point on the surface (where the curve equals 0%) to its lowest point (where the curve reaches 100%). The nomenclature and definition of bearing area ratio parameters are summarized in Table 5.

Bearing area ratio curves of a random rough surface are S-shaped, as illustrated in Figure 6. The reduced peak height *Spk* is the average height of the protrusion above the roughness core profile, which indicates the area that quickly wears out by relative motion. The reduced valley depth *Svk* is the average depth of the profile valleys projecting through the roughness core profile, which demonstrates the sub-surface that retains oil for lubrication. The core roughness depth *Sk* is the roughness profile excluding the fine protruding peaks and deep steep-sided valleys, which is the core depth of the functional surface during the lifetime of the surface. The material component *Smr1* is the percentage of the material ratio curve that coincides with the upper limit of the roughness core profile *Sk*. The material component *Smr2* is the percentage of the material ratio curve that coincides with the lower limit of the roughness core profile *Sk*. The pole height *Sxp* generally represents the height difference between the material ratio of 2.5% and the material ratio of 50%. The parameters *Spk* and *Svk* are each calculated as the height of the right-angle triangle, which is constructed to have the same area as the “peak area” or “valley area”, respectively. The right-angle triangle corresponding to the “peak area Area1” has *Smr1* as its base, and that corresponding to the “valley area Area2” has *Smr2* as its base.

### 3.3. Nanoindentation Tests Analysis

**Figure 7:**
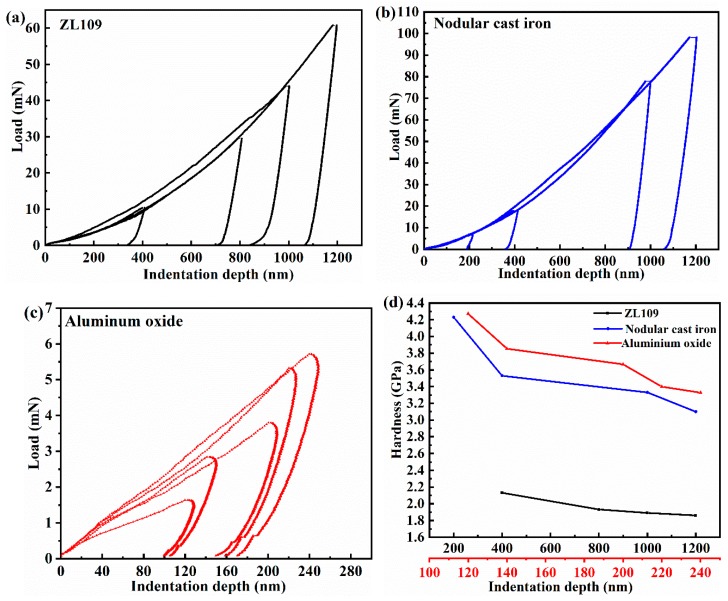
Load-unload curves dependence on the indentation depth for (**a**) ZL109; (**b**) nodular cast iron; and (**c**) aluminum oxide; (**d**) nanoindentation hardness.

The load-unload curve and superficial hardness over different indentation depths for ZL109, nodular cast iron, and aluminum oxide (black, blue, and red lines) are depicted in Figure 7. The phenomenon that the nanoindentation hardness decreases with the increasing maximum displacement is called size effect. The hardness of aluminum oxide increases by anodizing compared with that of ZL109, which is higher than that of nodular cast iron.

### 3.4. Frictional Coefficient

**Figure 8:**
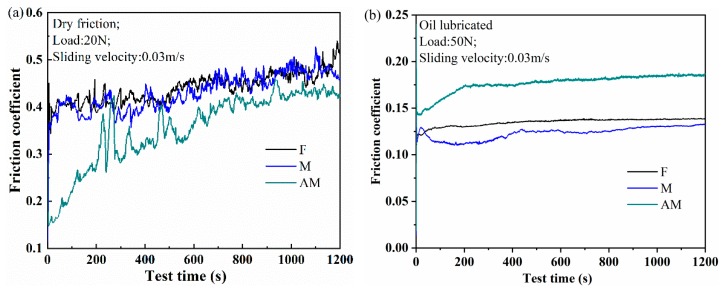
Frictional coefficients varying with running time under (**a**) dry sliding; (**b**) oil-lubricated conditions.

Figure 8 shows the plot of frictional coefficients varying with running time of 1200 s to elucidate the frictional property of F, M, and AM specimens under dry and oil-lubricated conditions. For sliding friction, both adhesion and ploughing components govern friction under dry sliding, while lubricated sliding can be characterized only by the ploughing component [30,37]. It is obvious that surface roughness controls friction process, since it fundamentally influences friction behavior. 

The friction coefficient of dry sliding conditions is illustrated in Figure 8a. Friction process of testing specimens can be divided into running-in and steady stages. Under steady state, the average frictional coefficient of flat specimens is 0.44. The mean friction coefficient of micro-textured samples is 0.43. For the anodizing micro-textured specimens, the friction coefficient is reduced compared with that of flat specimens. The average friction coefficient of AM samples is 0.37.

The friction coefficient of oil-lubricated conditions is shown in Figure 8b. The average friction coefficient of flat samples is 0.13 under steady stage. Moreover, the mean friction coefficient of micro-textured samples is 0.11, lower than that of flat specimens. This indicates that micro-textures enhance the frictional property through serving as lubricant reservoirs to generate secondary lubrication. However, the friction coefficient of anodizing micro-textured samples increases from 0.14 to the value of 0.17, which is higher than that of flat samples, and the mean friction coefficient of micro-textured specimens treated with anodizing is 0.16.

### 3.5. Discussions

#### 3.5.1. Influence of Bearing Area Ratio Curve and Relevant Roughness Parameters (*Sa, Sq, Spk, Svk, Sk, Smr1, Smr2*) under Dry Sliding Conditions

The two contributions, which are material property and surface topography, of two interacting specimens affect the frictional behavior under dry sliding tribological tests. In this research, the surface roughness and superficial hardness of micro-textured samples after anodizing increase compared with that of micro-textured specimens. The synergy between surface roughness and high superficial hardness of micro-textured samples after anodizing presents lower friction coefficient than that of merely micro-textured specimens.

Under dry sliding conditions, the frictional resistance can be expressed as the sum of two terms, the shearing and the ploughing process. According to Bowden and Tabor [22], the friction coefficient is given by

(1) $$F_{f}=A_{r}\tau_{s}+A_{p}\tau_{p},$$

(2) $$A_{r}=\frac{L}{H},$$

where *A_r_* is the real contact area, *A_p_* is the furrow area, *τ_s_* is the shear strength of a unit area, *τ_p_* is the furrow force of a unit area, *L* is the applied normal load, and *H* is the metallic hardness.

If the ploughing term is neglected, as the shearing term is the most important part which is responsible for metallic friction, the friction coefficient can be presented as:

(3) $$F_{f}=A_{r}\tau_{s},$$

The friction coefficient is closely related to the real contact area under dry sliding conditions. The real contact area is affected by the surface topography and superficial hardness of contact surfaces.

To investigate the influence of bearing area ratio curves on the tribological performance of testing samples, the bearing area ratio curves and friction real-time curves of flat, micro-textured, and anodizing micro-textured specimens are chosen for comparison, as shown in Figure 6 and Figure 8. A higher surface roughness (*Sa*, *Sq*) generally results in lower friction but longer distances to reach steady-state conditions under dry sliding conditions.

According to Zhu et al. [34], the dry sliding friction coefficient has a positive correlation with the bearing area ratio curves and its relative parameters. For the flat and micro-textured samples illustrated in Figure 8a, the smaller *Spk* and *Smr1* are, the faster the friction coefficient increases. In contrast, the larger *Spk* and *Smr1* are, the slower the friction coefficient rises, such as for anodizing micro-textured specimens. Compared with anodizing micro-textured samples, the *Sk* of flat and micro-textured specimens are the smallest. Consequently, the changes in the friction coefficient of flat and micro-textured samples are accordingly minimal. Therefore, the smaller the value of *Sk*, the faster the friction coefficient reaches the steady state, and the running time to steady-state conditions tends to become shorter under dry sliding conditions. A smaller ratio of real contact area to nominal contact area is presented with steeper bearing area ratio curve for larger *K*, *Spk*, *Sk*, and *Svk* values of anodizing micro-textured samples, and the high superficial hardness of anodizing micro-textured samples reduces the real contact area as well. Consequently, anodizing micro-textured samples present the lowest friction coefficient under dry sliding friction.

In the case of dry sliding, friction coefficient curves of anodizing micro-textured samples exhibit a certain degree of oscillations, as seen in Figure 8a. Menezes et al. [37] attribute these oscillations to the stick-slip effect related to friction process development and adhesive component. They report that surface roughness does not affect the amplitude of these oscillations significantly, but that they are mainly influenced by the ploughing component of the friction. Stick-slip phenomenon is also usually related to the transfer of materials in contact.

**Figure 9:**
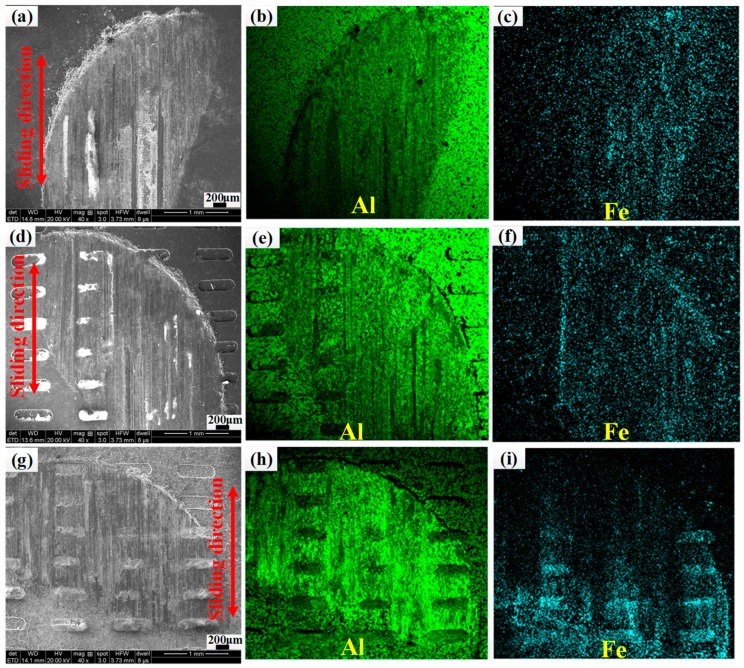
SEM micrographs of flat, micro-textured, and anodizing micro-textured specimens (**a**,**d**,**g**) and EDS element mapping data of (**b**,**e**,**h**) Al, (**c**,**f**,**i**) Fe.

SEM images and the EDS element mapping of worn surfaces (Figure 9) are given to elucidate the element transformation between the two contact surfaces under dry friction conditions. The arrows indicate the sliding direction of the plate relative to the pin. The lime and dark-cyan phases indicate Fe and Al elements, respectively. Noticeable transfer of Fe elements from nodular cast iron samples onto the disc is observed on the anodizing micro-textured samples, which is consistent with the reason why the friction coefficient of anodizing micro-textured samples exhibit high oscillations.

#### 3.5.2. Influence of Bearing Area Ratio Curve and Relevant Roughness Parameters (*Sa, Sq, Spk, Svk, Sk, Smr1, Smr2*) under Oil-Lubricated Conditions

The three contributions to the friction coefficient are surface roughness, superficial hardness, and lubricant property under oil-lubricated conditions. As concluded in reference [31], the friction coefficient is lower when roughness is low for the lubricated test. Under oil-lubricated conditions, the large surface roughness increases the friction coefficient of micro-textured specimens after anodizing, even the high superficial hardness of alumina films. The large surface roughness presents a negative effect to form oil film to reduce the friction under oil-lubricated conditions.

Under boundary lubrication, the overall friction arises from the metallic contact as well as the shear in the film. The friction coefficient is

(4) $$f=\alpha f_{m}+(1-\alpha )f_{l},$$

where *α* is the fractional film defect, which is defined as the fraction of real area that is metallic, *f_m_* is the friction coefficient for metallic junctions, and *f_l_* is the friction coefficient for boundary lubrication.

Under the large normal load and the same sliding speed conditions, the average friction coefficients are several times lower than those of dry friction due to the presence of the lubricant film that bears most of the load. Comparing the surface roughness parameters with the friction coefficient, it can be observed that the lubricated friction coefficient generally increases with the *Sa* and *Sq* values, which is the opposite of the case for dry sliding. Moreover, the lubricated sliding distances to steady-state conditions are, in principle, longer for rougher surfaces.

Under oil-lubricated conditions, friction coefficient has a negative correlation with the bearing area ratio curves and its relative parameters. The *Spk*, *Smr1*, and *Sk* values of flat and micro-textured sample are smaller than those of anodizing micro-textured specimens, which results in shorter sliding distance to steady-state friction conditions. Consequently, for lubricated friction, the smaller the *Spk*, *Smr1*, and *Sk* values are, the shorter the running time to steady-state friction conditions.

The macro slope *K* of the material ratio curve increases with *Sa* and *Sq*, as illustrated in Figure 6. The material ratio curve becomes much steeper for larger values of *K*, *Spk*, *Sk*, and *Svk*, which results in a smaller ratio of real contact area to nominal contact area and a larger average distance between the surfaces. Small ratio of real contact area to nominal contact area is beneficial to reduce friction coefficient under dry sliding conditions. However, the increasing large average distance is harmful to reduce the the friction under oil-lubricated conditions due to the decreasing film thickness ratio *h_min_*/*σ*. The small film thickness ratio will decrease the lubricated film bearing capacity, increase the load ratio of the interference region, and ultimately result in exacerbated wear and an increased friction coefficient [38,39,40]. The *K*, *Spk*, *Sk*, and *Svk* values of anodizing micro-textured specimens are larger than those of flat and micro-textured samples, which causes its mean friction coefficient to become the largest due to the lowest bearing capacity of lubricating film. Consequently, for lubricated friction, the larger the *Spk*, *Smr1*, and *Sk* values are, the higher the average friction coefficient is. 

## 4. Conclusions

In this study, the effects of surface roughness on the friction property of flat, micro-textured, and anodizing micro-textured samples were investigated under dry sliding and oil-lubricated conditions. The conclusions can be drawn as follows:

(1) The dry friction coefficient has a positive correlation with the bearing area ratio, which decreases with larger surface roughness parameters of *K*, *Spk*, *Sk*, and *Svk*. The synergy between surface roughness and high superficial hardness of micro-textured samples after anodizing presents a lower friction coefficient than that of flat and merely micro-textured specimens under dry friction.
(2) The large surface roughness parameters of *K*, *Spk*, *Sk*, and *Svk* present negative effects on forming oil film to reduce the friction under oil-lubricated conditions. The anodizing micro-textured samples present a larger oil-lubricated friction coefficient than the flat and micro-textured specimens.
(3) The running time to steady-state friction conditions tends to become longer with the increase in surface roughness parameters of *Spk*, *Smr1*, and *Sk* under both dry and oil-lubricated sliding conditions.

## Figures and Tables

**Table 1:** Chemical composition of ZL019.

Element	Si	Fe	Cu	Mg	Ni	Al
Content (%)	11.1	0.77	0.45	0.51	0.31	Residual

**Table 2:** Cutting parameters of fabricating micro-textures.

Cutting Amount	Spindle Speed (r/min)	Feed Rate (mm/min)	Axial Depth of Cut (μm)
Parameters	20,000	60	10

**Table 3:** Testing parameters of nanoindentation.

Testing Materials	Maximum Displacement (nm)	Spacing (μm)
ZL109	400, 800, 1000, 1200	20
Nodular cast iron	200, 400, 1000, 1200	20
Aluminum oxide	120, 140, 200, 220, 240	10

**Table 4:** Surface roughness of test samples.

Surface Roughness (μm)	F	M	AM
*Sa*	0.92 ± 0.05	0.89 ± 0.05	1.81 ± 0.05
*Sq*	1.1 ± 0.1	1.1 ± 0.1	2.5 ± 0.1
*Ssk*	0.38 ± 0.05	0.09 ± 0.05	−0.05 ± 0.05
*Sku*	3.2 ± 0.1	3.5 ± 0.1	6.3 ± 0.1
*Sk*	2.8 ± 0.1	2.7 ± 0.1	4.6 ± 0.1
*Spk*	1.4 ± 0.1	1.3 ± 0.1	3.9 ± 0.1
*Svk*	0.9 ± 0.1	1.1 ± 0.1	3.8 ± 0.1
*Sp*	4.8 ± 0.4	5.0 ± 0.4	14.0 ± 0.4
*Smr1*	13.1 ± 1.5	10.6 ± 1.5	14.2 ± 1.5
*Smr2*	91.7 ± 1.5	89.2 ± 1.5	88.3 ± 1.5

**Table 5:** Nomenclature and definition of bearing area ratio parameters.

Material Ratio Parameters	Definitions
*Spk*	Average height of the protruding peaks above the roughness core profile
*Svk*	Average depth of the profile valleys projecting through the roughness core profile
*Sk*	Depth of the roughness core profile
*Sxp*	The height difference between the load area ratio of 2.5% and the load area ratio of 50%
*Smr1*	Level, in percent, determined for the intersection line which separates the protruding peaks from the roughness core profile
*Smr2*	Level, in percent, determined for the intersection line which separates the deep peaks from the roughness core profile
